# Thank you to *The Lancet Regional Health - Southeast Asia*’s clinical and statistical peer reviewers in 2022

**DOI:** 10.1016/j.lansea.2023.100159

**Published:** 2023-02-06

Mohammed Aal

Misra Abdulahi

Mohd-Asyraaf Abdul-Kadir

Jabir Abdullakutty

Ishanya Abeyagunawardena

Gerard Abou Jaoude

Ivo Abraham

Krishna Acharya

Venkata Suresh Adapa

Bipin Adhikari

Deepika Adhikari

Hassan Sami Adnan

Akhilesh Agarwal

Sandip Agarwal

Nima Aghaeepour

Umang Agrawal

Rodrigo Aguayo-Romero

Indu B Ahluwalia

Tarunveer S Ahluwalia

Firoz Ahmad

Shafqat Ahmad

Suleiman Idris Ahmad

Anas Ahmed

Bilal Ahmed

Md Montasir Ahmed

Taha Ahmed

Tahmeed Ahmed

Joel Aik

Samuel Akech

Radha Rama Devi Akella

Kafil Akhtar

Gulali Aktas

Fahmida Akter

Taghreed Al Haidari

Md Tauqeer Alam

Mohammed Alam

Alexandre Alanio

Fatima Safira Alatas

Arm Rmm Ali

Sajid Ali

Musheer Aljaberi

Mohamad Aljofan

Fatima Almaghrabi

Ricardo Almendra

Maria Sofia V Amarra

Rafi Amir-ud-Din

Ankit Anand

Sharon Anavi-Goffer

Roy Anderson

Takeshi Annoura

Kathleen Antony

Sumadi L Anwar

S M Yasir Arafat

Rajeev Aravindakshan

Aleksandr Y Aravkin

Gaston Ares

Eggi Arguni

Rahul Arora

Hafsa Arshad

Priti Arun

Uzma Ashraf

Sadhana Awasthi

Win Aye

Ahmed Y Azzam

Karan Babbar

Giridhara Babu

Christine M Bachman

Ahmed Suparno Bahar Moni

Anurima Baidya

Vidya Baliga

Tarık Balkan

Susovan Banerjee

Ali Haider Bangash

Aditya Bardia

Erez Bar-Haim

Ivan Barilar

Abdul Basit

Buddha Basnyat

Hasan Basri

Saurav Basu

Katherine Battle

Neha Batura

Sunita Bavanandan

Purva Bavikar

Arjun Bedi

Sarah Bernard

Anna Bershteyn

Sapna Bharti

Rahul C Bhoyar

Zulfiqar Bhutta

Fadil Bidmos

Ashna Biju

Andreas L Birkenfeld

Branko Bobić

Giorgio Bogani

Georg Böhmig

Srinivasa Reddy Bonam

Kaushik Bose

John Bradley

Stephane Bretagne

Marcella Broccia

Meredith Brooks

Anitha C T

Andrea Cabibbe

Miao Cai

Qingxian Cai

Harlan Campbell

Jackie Campbell

Jane Y Carter

Marisa Casale

Rodrigo Cataldi

Silvia Maria Baeta Cavalcanti

Panjaporn Chaichana

Chanapai Chaiyakulsil

Joy Chakma

Suman Chakrabarty

Pinaki Chakraborty

Santam Chakraborty

Kam Wa Chan

Prabhat Chand

Vishal Chander

Arunah Chandran

Dilip Chandrasekhar

Debajyoti Chatterjee

Arun Chaturvedi

Pankaj Chaturvedi

Khushboo Chauhan

William Checkley

Chung-Hwan Chen

Runsen Chen

Yongjie Chen

Zhishui Chen

Li Cheung

Shakunatala A Chhabra

Kamal Kishore Chopra

Om Choudhary

Pradip Chouhan

Mohiuddin Ahsanul Kabir Chowdhury

Thomas F Clasen

Tammy Clifford

Andrew Copas

Rosie Cornish

Marcelo Fernandes Costa

Kathrin Cresswell

Simone Crouch

Caroline Daccache

Ann Daly

Manoja Das

Sourav Das

Soumitra Shankar Datta

Harsh S Dave

Haiima Dawood

Bas de Laat

Santiago De Ossorno Garcia

Varuni de Silva

Mariana de Souza

Tom Decroo

Smita Deshpande

Arnab Dey

Manish Dhawan

Nadia Diamond-Smith

James Diffey

Jigeeshu Divatia

Priyanka Dixit

Warren Dodd

Shaantanu Donde

Yanhui Dong

Gowri Dorairajan

Sithar Dorjee

Thinley Dorji

Ibrahim Duah

Sunita Dubey

Dorothea Dumuid

Shanta K Dutta

Elizabeth Dylke

Emmanuel Dziwornu

Joel Ellwanger

Patrick Hoang Vu Eozenou

Mohammed Eslam

Sara Estecha Querol

Paolo Eusebi

Ugochukwu Eze

Kaja Falkenhain

Eric Faragher

Forough Farrokhyar

Muhammad Abdullah Fazi

Laura Ferguson

Henk Folmer

Anna Freni Sterrantino

María Guadalupe Frías-De-León

Liang Fu

Ayataka Fujimoto

Silvano Gallus

Sumanth Gandra

Mahendra Garg

Suneela Garg

Tushar Garg

Gladwell Koku Gathecha

SubbaRao Gavaravarapu

Ezra Gayawan

Anice George

Shishirendu Ghosal

Pilar Giraldo

Javier Gisbert

Anette P Gjesing

Sonu Goel

Michael Gold

Eduardo Gomez

Gyanendra Gongal

Vikram Gota

John Gregson

Karen Grépin

Kamal Gulati

Sheffali Gulati

Qinghai Guo

Piyush Gupta

Rajeev Gupta

Ribhav Gupta

Yashdeep Gupta

Kapil Gururangan

Narayan Gyawali

Nyssa Hadgraft

Firdaus Hafidz

Saeed Hamid

Claudia Hanson

Timotius Hariyanto

Diane Harper

Joseph Harris

M Tasdik Hasan

Mashfiqul Hasan

Ben Haseen

Khezar Hayat

Yulei He

Linnea Hedman

Nicholas Heming

Julia B Hennermann

Fred Hersch

Thaddeus Herzog

Graham Hitman

Xiumei Hong

Surat Hongsibsong

Mohammad Hoque

Ismail Hosen

Ahmed Hossain

Motaher Hossain

Saima Wazed Hossain

John Howard

Qiuhao Huang

Khadija Nuzhat Humayun

Keith Humphreys

Kumaravel Ilangovan

Sushrut Ingawale

Ana-Maria Iosif

Siara Isaac

Taisuke Ishii

Khaleda Islam

Md Islam

A Heri Iswanto

Takuya Iwamura

Karthikeyan Iyengar

Priya Iyer

Bart Jacobs

Uday Mahadeo Jadhav

Mayank Jain

Asif Jamal

Krishnamurthy Jayanna

Pabitra Jena

Aleksandra Jezela-Stanek

Wenqing Jiang

Fengshi Jing

Taufique Joarder

William Joe

Srujana Joga

Sajjakaj Jomnonkwao

Himanshu Joshi

Madhvi N Joshi

Elmar Joura

Deven Juneja

Shaji K S

Seyram Kaali

Conrad B Kabali

Furqan Kabir

Manoj Kalita

Vijaya Kancherla

Osamu Kaneko

Madhuri Kanitkar

Shuvra Kanti Dey

Shantanu Kar

Sujita Kar

Nasser Karimi

Tilakavati Karupaiah

Rakesh Katna

Tomohiro Katsuta

Harpreet Kaur

Jagdish Kaur

Japneet Kaur

Prabhdeep Kaur

Ashish K C

Jeremy Keenan

Masoud Keikha

Sherrie Kelly

Mark Kelson

Zoe Kelson

Mohammad Ibrahim Khalil

Naila Khan

Shakir Khan

Rajeshwar Khandare

Bidita Khandelwal

Narges Khanjani

Ali Khashan

Mohsin Khurshid

Jung-Sun Kim

Andre Konski

Evangelos Kontopantelis

Soewarta Kosen

Zisis Kozlakidis

Elke Olivia Kreps

Bhargav Krishna

Anand Krishnan

Ganapathy Krishnan

G V Krishnaveni

Evangelos Kritsotakis

Vidushi Kulshrestha

Abhinendra Kumar

Ajit Kumar

Kaushalendra Kumar

Manish Kumar

Rajat Kumar

S Kumar

Sandeep Kumar

Anton Kunst

Nia Kurniati

Yohannes Kurniawan

Karishma Krishna Kurup

Vivek Kute

Vellappillil Kutty

Chan-Young Kwon

Myat Phone Kyaw

Riyadh Lafta

Sham Lal

Mamatha Murad Lala

Bradley J Langford

Mohd Talib Latif

Anthony Laverty

Avula Laxmaiah

Gilbert Lazarus

Carlo Lazzaro

Emilie Le Rhun

Andrew Leather

Eric Kam-Pui Lee

Seungyup Lee

Mondher Letaief

Sanjiv Lewin

Hanming Li

Shizhu Li

Zijian Li

Khin Lin

Yinan Lin

Mark Litaker

Hanruo Liu

Zhaolan Liu

Carmelo Loinaz-Segurola

Jin Lu

Beatrice Kemunto MacHini

Pranab Mahapatra

Asri Maharani

Ruby Maharjan

Surakameth Mahasirimongkol

Geofrey Makenga

Kanika Malik

Supriya Mallick

Helena Maltezou

Mohammed Mamun

Manikandan Mani

Raj Kumar Mani

Adil Maqbool

Alberto Marcacuzco

Ana Marques

Adolfo Martinez-Valle

Ryuichi Mashima

Shahril Azian Haji Masrom

Sri Masyeni

Beela Sarah Mathew

Prashant Mathur

Fiona Matthews

Anne Thushara Matthias

Sarah McIntyre

Rachael McLean

M L Meena

Saurabh Mehta

Geetha Menon

Indrapal Ishwarji Meshram

Stefan Michiels

I Gede Nyoman Mindra Jaya

Wai Kit Ming

Jose Joaquin Mira-Solves

Patrick Mirindi

Anurag Mishra

Baijayantimala Mishra

Jannatul Mawa Misti

Satoshi Mitarai

Diwakar Mohan

Indu Mohan

Viswanathan Mohan

Sreelekshmy Mohandas

Bidhu Mohanti

Sanjay Mohanty

Prasanta Mohapatra

Ranjan K Mohapatra

Sanjeeb Mohapatra

César Molina

Nwe Oo Mon

Nachiket Mor

Mary Ann A Muckaden

Debjani Mueller

Partha Sarathi Mukherjee

Satinath Mukhopadhyay

Siuli Mukhopadhyaya

Ivan Müller

Sanjay Kumar Munda

V R Muraleedharan

Manoj Murhekar

Srinivas Murthy

Siti Muslimatun

Prashanth N Srinivas

Sudhir Nair

Tiny Nair

Geeta Sowmya Narayanan

Sudirman Nasir

Gheyath Nasrallah

Jyotiman Nath

Sheila Nathan

Fahd Naufal

Bijoor Shívananda Nayak

Ahsana Nazish

Shekhar Neema

Sutapa Neogi

Dinesh Neupane

Joshua Ng-Kamstra

Michele Nguyen

Emily Nightingale

Ngozi M Nnam

John Norrie

Bernard North

Dalton Norwood

Nuning Nuraini

Achmad Nurmandi

Kieran O'Brien

Alberto Ochoa-Zezzatti

Tatsuya Ohno

Ayako Okuyama

Catherine Oldenburg

Mihai Oltean

Isao Oze

Ramachandran Padmavati

Nonglak Pagaiya

Madhukar Pai

Muralidhar V Pai

Saibal Kumar Pal

José Miguel Palacios-Jaraquemada

Prasit Palittapongarnpim

Sonali H Palkar

Kishor Pandey

Prashant Kumar Pandey

Jeyaraj Pandian

Dimitrios Papadimitriou

Andee Cooper Parks

Laurie Parsons

Ayodhia Pasaribu

Shivani Patel

Jay R Patwa

Piero Pavone

Dharshani Pearson

Nicole Pecora

Michael Peters

Simone Pettigrew

Praphan Phanuphak

Nishad Plakkal

Abel Po-Hao Huang

Yashashwi Pokharel

Antonio Portolés

Wiraporn Pothisiri

Kalpana Poudel-Tandukar

Pradeep Kumar

Manas Ranjan Pradhan

Ravi Prakash

Robin Prescott

Shankar Prinja

Sugyanta Priyadarshini

Ari Probandari

Audrey Prost

Sher Bahadur Pun

Saravanakumar Puthupalayam Kaliappan

Nina Putri

Zahidul Quayyum

Dario Rahelić

Md Mostafizur Rahman

Rajesh Kumar Rai

Sree Bhushan Raju

Astha Ramaiya

Lakshmy Ramakrishnan

Jaishree Raman

Vijayalakshmi Ramshankar

Jonas Ranstam

Ananth Rao

R Shyama Prasad Rao

Raghuram Rao

Chaturbhuj Rathore

Prima Dhewi Ratrikaningtyas

Sofia Belo Ravara

Sudhir Kumar Rawal

Shruta Rawat

K Srinath Reddy

Torsten Risør

Eric Rogier

Winsley Rose

Peter Rossing

Isobel Routledge

Ria Roy

Paisan Ruamviboonsuk

Vedantam Rupa

Mihir Prafulbhai Rupani

Banshi Saboo

Harshpal Sachdev

Mahipal Singh Sachdev

Marc Saez

Jaya Prakash Sahoo

Jitendra Sahu

Kamal Sahu

Eiko Saito

Sho Saito

Avanish Saklani

Abolfazl Salari

Sheikh Saleem

Mohammad Reza Salehi

Oscar Daniel Salomon

Shrikant Salve

Lubna Samad

Elena Samonova

Gadey Sampath

Aamer Sandoo

Marsha Sinditia Santoso

Kallur Saraswathy

Malabika Sarker

Usha Sarma

Gargi Sarode

Sachin Chakradhar Sarode

Shashi Saroj

Nadimpally B Sarojini

Muttukrishna Sarvananthan

Jitendra Pal Singh Sawhney

Renu Saxena

Abu Sayeed

Stephen Schensul

Joergen Schlundt

Jennifer Seager

Giada Sebastiani

Inderpaul Sehgal

Sumithra Selvam

Kalaiselvi Selvaraj

Lalith Senarathna

Shekhar P Seshadri

Ery Setiawan

Dheeraj Shah

Duru Shah

Yogendra Shah

P Ravi Shankar

Coimbatore Subramanian Shanthi Rani

Indar Sharawat

Ashima Sharma

Dheeraj Sharma

Megha Sharma

Nandini K Sharma

Vijay sharma

Aziz Sheikh

Yun Shen

Kottacherry Trivikrama Shenoy

Zumin Shi

Sachin Shinde

Poonam Varma Shivkumar

Saurabh Shrivastava

Ching Sin Siau

Kamran Siddiqi

Derrick Silove

Ebby George Simon

Manish Kumar Singh

Navneet Singh

Prashant Kumar Singh

Shalini Singh

Vikas Singh

Abhinav Sinha

Animesh Sinha

Bireshwar Sinha

Sathish Kumar Sivasankaran

Ted R Smith

Wing Yee So

Anandina Irmagita Soegyanto

Sunghwan Sohn

Vandana Soni

Francisca Beatriz De Melo Sousa

Somashekhar SP

Gianfranco Spalletta

Nikhil Srinivasapura Venkateshmurthy

Ankur Srivastava

Shobhit Srivastava

Swati Srivastava

Massimo Stafoggia

Pasquale Stefanizzi

Leon Straker

Deepak Subedi

Alok Sud

Faisal Sultan

Faisal Sultan

Mahesh Sultania

Xiaojie Sun

Avinash K Sunny

Nikita Shiraz Surani

Auliya A Suwantika

Thomas A Swain

Hayato Tada

Ajay Tandon

Rohit Tandon

Sarmila Tandukar

Julian Tang

Marjan Tariverdi

Alessandro Tarozzi

Alvin Kuowei Tay

Yassen Tcholakov

Jaap ten Oever

Krishna K Thakur

Shalini Thakur

Dipu Thareparambil Sathyapalan

Kannan Thiruvengadam

Ramachandran Thiruvengadam

George Thurston

Mangesh Tiwaskar

Kelvin To

Tashi Tobgay

Hidenori Toyoda

Yuda Turana

Bhishma Tyagi

Cesar Ugarte-Gil

Dhamodharan Umapathy

Ambika Gopalakrishnan Unnikrishnan

Sundeep Upadhyaya

Michael F Urban

Girija Vaidyanathan

Mahdi Vajdi

Eddy van Doorslaer

Onno C P van Schayck

Jithin Varghese

Rajani Ved

Chitra Venkateswaran

Peter Ventevogel

Francesco Venturelli

James Venturini

Eliseu Verly

Madhur Verma

Yogesh Verma

Aishwarya Lakshmi Vidyasagaran

Vijay Viswanathan

Jadranka Vraneković

Girija Narendrakumar Wagh

Nidhi Wali

Gurpreet S Wander

Jiguang Wang

Reynold G Washington

Guenter Weiss

Paul Weiss

Xue Wen

Justin White

Phoebe Williams

Phoebe Williams

Yuniardini Septorini Wimardhani

Pattanee Winichagoon

Woranan Witthayapipopsakul

Johann Wojta

Grace Wong

Vincent Wong

Arthur W Wu

Anita Yadav

Pragya Yadav

Chittaranjan Yajnik

Weihui Yan

Sandhya Kanaka Yatirajula

Edmund Yeboah

Ritthideach Yorsaeng

Masoud Yousefi

Evy Yunihastuti

Rabaab Zahra

Wei Zhang

Li-Ping Zou

Juliana Zuliani

